# The Effect of UV Aging on Antimicrobial and Mechanical Properties of PLA Films with Incorporated Zinc Oxide Nanoparticles

**DOI:** 10.3390/ijerph15040794

**Published:** 2018-04-18

**Authors:** Małgorzata Mizielińska, Urszula Kowalska, Michał Jarosz, Patrycja Sumińska, Nicolas Landercy, Emmanuel Duquesne

**Affiliations:** 1 Center of Bioimmobilisation and Innovative Packaging Materials, Faculty of Food Sciences and Fisheries, West Pomeranian University of Technology Szczecin, Janickiego 35, Szczecin 71-270, Poland; urszula.kowalska@zut.edu.pl (U.K.); michal.jarosz@zut.edu.pl (M.J.); patrycja.suminska@zut.edu.pl (P.S.); 2 Materianova, Avenue Nicolas Copernic, 1, 7000 Mons, Belgium; nicolas.landercy@materianova.be (N.L.); emmanuel.duquesne@materianova.be (E.D.)

**Keywords:** ZnO nanoparticles, antibacterial, antimicrobial properties, mechanical properties

## Abstract

The aim of this study was to examine the influence of accelerated UV-aging on the activity against chosen microorganisms and the mechanical properties of poly-lactic acid (PLA) films enhanced with ZnO nanoparticles. The pure PLA films and tri-layered PLAZnO1%/PLA/PLAZnO1% films of 150 µm thickness were extruded. The samples were treated with UV-A and Q-SUN irradiation. After irradiation the antimicrobial activity and mechanical properties of the films were analyzed. The results of the study demonstrated that PLA films did not inhibit the growth of *Staphylococcus aureus*, *Bacillus cereus*, *Escherichia coli*, *Bacillus atrophaeus*, and *Candida albicans* cells. PLA films with incorporated zinc oxide nanoparticles decreased the number of analyzed microorganisms. Accelerated UV aging had no negative effect on the activity of the film containing nano-ZnO against Gram-positive bacteria, but it influenced the activity against Gram-negative cells and *C. albicans*. Q-SUN irradiation decreased the antimicrobial effect of films with incorporated nanoparticles against *B. cereus*. UV-A and Q-UV irradiation did not influence the mechanical properties of PLA films containing incorporated ZnO nanoparticles.

## 1. Introduction

The main function of food packaging is to maintain the quality and safety of food products during storage and transportation and to extend the shelf-life of the packed products by preventing unfavorable factors or conditions, such as microorganisms which cause spoilage, chemical contaminants, oxygen, moisture, or UV light [1]. The incidence of increased bacterial foodborne infection, bacterial antibiotic resistance or the emergence of new bacterial mutations, are a global health hazard to humans. It has been shown that *Shigella flexneri* causes 1.5 million deaths annually, due to contaminated food and drinks, as reported by Kotloff, K.L. et al. [2]. The number of Shigellosis was found to be 164.7 million, of which 163.2 million were in developing countries (with 1.1 million deaths) and 1.5 million in industrialized countries. A total of 69% of all episodes, and 61% of all deaths, attributable to shigellosis involved children under five years of age. The median percentages of isolates of *S. flexneri*, *Shigella sonnei*, *Shigella boydii*, and *Shigella dysenteriae* were 60%, 15%, 6%, and 6% (30% of *S. dysenteriae* cases were type 1) in developing countries, respectively, and 16%, 77%, 2%, and 1% in industrialized countries.

Thus, the development of novel antibacterial agents against bacteria, such as *Escherichia coli* O157:H7, *Campylobacter jejuni*, *Staphylococcus aureus*, *Pseudomonas aeruginosa, Enterococcus faecalis*, *Salmonella types*, and *Clostridium perfringens*, has become a top priority [3]. The antibacterial activity of many active substances, such as essential oils, plant extracts, and preservatives, have received significant global interest worldwide. Many microorganisms exist in sizes that range from hundreds of nanometers to tens of micrometers long. The growth of microorganism cells may be decreased, or even inhibited, particularly through the implementation of nanotechnology to synthesize particles at the nanometer scale [3,4,5,6]. The addition of ZnO nanoparticles into packaging materials is considered highly promising, as it may inhibit microorganisms and improve food quality. It has been clearly shown that the use of the nanoparticles introduced into the polymeric matrix or used as an additive to a coating can increase the shelf-life of packaged product by inhibiting of the growth of foodborne pathogens [4,5,6,7,8,9]. A highly important advantage of ZnO nanoparticles is their acceptance as a safe additive (GRAS) by the United States Food and Drug Administration (USFDA, 21CFR182.8991) [4,5,6,10]. Nanoparticles of ZnO may inhibit Gram-positive and Gram-negative bacteria and these particles may decrease the number of the spores [11,12,13], yeasts, and fungi [14]. This means that nanoparticles have offer strong antimicrobial activity against a wide spectrum of microorganisms, with the activity depending on their surface, shape, and concentration [5]. Many reports have shown that particle size influences the antibacterial activity of ZnO nanoparticles. It was found that the antibacterial activity of ZnO nanoparticles increased as the particle size decreased. Especially, size-dependent activity was observed at a range of 100–800 nm in *S. aureus* and *E. coli* [15]. Several mechanisms relating to the antimicrobial activity of ZnO nanoparticles were described, such as cell wall damage, the generation of superoxide anions, hydroxyl radicals, hydrogen peroxide, as well as the penetration of the cell envelope. The effect of nanoparticle morphology and crystalline structure on antimicrobial activity was also noted. Nano-sized ZnO can interact with the bacterial surface and/or with the bacterial core when it enters the cell. It was found that the surface area of the ZnO nanoparticle was the greatest when it was a hexagonal pyramid, which results in a more effective antibacterial action than in the case of star and hexagonal rod-shaped ZnO [3,16,17]. ZnO nanoparticles interact with cells, membranes, proteins, and DNA, establishing a nano-bio interface. The functional aspects of the interface depend on colloidal forces, as well as physicochemical interactions. ZnO surface potential induces an electrostatic field around it, which, in turn, reorients the local water population up to a certain depth into the bulk, though this depends upon the electrostatic field strength. This causes a rearrangement of the whole biological mechanisms, like protein folding, membrane dynamics, and enzyme catalysis. Additionally, metallic nanoparticles with photocatalytic properties result in a non-specific inhibition of microbial growth, which come about as a result of reactive oxygen species (ROS) generation as nanoparticles come into contact with radiation or media. The energy band gap of nano-ZnO is so small that, on radiation absorption, excited nanoparticle electrons cause cascade reactions of ROS production [18].

Due to its high antibacterial ability, ZnO nanoparticles have been incorporated into synthetic polymers, such as low-density polyethylene (LDPE), isotactic polypropylene (iPP), and polyamide (PA) [16]. Though synthetic plastic packaging materials have been widely used for the packaging of various types of food, they cause a serious environmental problem since they do not degrade in the environment after use. Ideal biodegradable packaging materials have to be obtained from renewable biological resources [1]. Metal oxide nanoparticles may be incorporated not only into the synthetic polymers, but may also be introduced into biopolymers, such as polylactic acid (PLA) [16]. They can also be added into polymer coating layers in the application of active packaging [6,14,17,18,19,20,21,22,23]. PLA is one of the most important bio-based polyesters, derived from a lactic acid monomer that is obtained from the fermentation of starch or other polysaccharide sources. PLA may also be synthesized using food industry by-products as a carbon source [24,25]. Polylactic acid films have significant potential for the packaging industry due to the yield of stiff films which offer high transparency and can be readily processed using available production technologies. Unfortunately these materials also have a number of shortcomings, such as poor thermal properties, toughness, and water vapor and gas barrier properties that are inferior to those of conventional petroleum-based polymers. It is would be possible to make PLA much more competitive to synthetic polymer films by improving its properties. One of the most important ideas is to add active substances, such as ZnO nanoparticles, that may improve the antimicrobial and mechanical properties of PLA [25,26].

To impart new functionalities to PLA-based antimicrobial packaging materials, some suitable nanoparticles, such as TiO_2_, Ag, or ZnO, may be incorporated within the PLA matrix [6,24,25,26,27,28]. In general, packaging should extend the shelf life of the food product through a decrease in the number of microorganisms or by growth inhibition. The packaging material should function during storage, meaning it should offer sufficient resistance against UV aging or be shielded against ultraviolet light by the additive.

UV radiation may contribute to the deterioration in the physico-mechanical, optical, and antimicrobial properties of biopolymer-based packaging materials. The introduction of antimicrobials that are not resistant to UV irradiation into PLA matrix may decrease packaging effectiveness on UV radiation, while the addition of an antimicrobial into the biopolymer matrix that is not sensitive to UV aging, or the addition of the antimicrobial with shielding properties, may prevent this decrease. Among metals and metal oxides, ZnO was found to be an ideal antimicrobial substance able to block UV radiation and improve packaging performance, which includes mechanical properties. It has been shown that ZnO in nano-form could be used as an ultraviolet light absorber that exhibited chemical stability under radiation. ZnO nanoparticles were incorporated with all types of unplasticized polymers at up to 2% weight, and were intended for contact with all types of foodstuffs for long-term storage. The addition of ZnO nanoparticles into the polymer matrix may improve UV shielding of all biopolymer-based films and ZnO in nano-form, and may even protect its antimicrobial effectiveness [5,6,29,30].

The consumption of fresh-cut vegetables and fruits became very popular over the last few decades. Consumer interest in healthy, nutritious diets has led to a change in lifestyle. Strawberries or fresh-cut apples have recently become popular due to their unique, highly desirable flavor, and because they are rich in polyphenols, anthocyanins, vitamins, and other bioactive compounds. However, the preservation of fresh-cut apples or strawberries is difficult work because they undergo rapid deteriorative processes, which lead to fruit decay. The fruit has a short shelf life due to enzymatic reaction, tissue softening, water loss, and microbial growth. Cold temperatures and modified atmosphere can increase the storage time of the fruit. Post-harvest losses of cut apples and strawberries are more serious at room temperature. These fruits may be sold at a market, where they are exposed to environmental conditions. In order to extend fruit shelf-life and improve the microbial purity of freshly-cut apple and strawberries, active PLA films with antimicrobial properties can be applied. Light is of primary importance with regards to antimicrobial film deactivation. The incorporation of the ZnO nanoparticles as UV resistant, antimicrobial agents within the PLA matrix may protect the packaging, and the fruits themselves, during storage at room temperature [24,31].

The aim of this study was to examine the influence of accelerated UV-A and Q-SUN irradiation (UV aging) on mechanical properties and the effectiveness of ZnO nanoparticle enhanced PLA films against a number of selected microorganisms.

## 2. Materials and Methods

### 2.1. Materials

The microorganisms that were used in this research work were obtained (bought) from a collection from the Leibniz Institute DSMZ (Deutsche Sammlung von Mikroorganismen und Zellkulturen) and from an American Type Culture Collection (ATCC). There were: *Staphylococcus aureus* DSMZ 346, *Bacillus atrophaeus* DSM 675 IZT, *Bacillus cereus* ATCC 14579, *Escherichia coli* DSMZ 498, and *Candida albicans* DSMZ 2566.

To examine the activity of PLA films against analyzed microorganisms, Triptic Soy Broth (TSB), Triptic Soy Agar (TSA), and Sabouraud media (Merck, Darmstadt, Germany) were chosen. All media were weighed according to the manufacturer’s (Merck) instructions, suspended in 1000 mL of distilled water, and autoclaved at 121 °C for 15 min.

Poly(l,l-lactide)—hereafter called PLA—supplied by NatureWorks LLC, was a grade designed for the extrusion of films (4032D) with *M*_n_ (molecular mass) =  133,000, dispersity (D), *M*_w_/*M*_n_  =  1.94 (*M*_w_ and *M*_n_ being, respectively, weight- and number-average molar masses expressed in polystyrene equivalent), whereas according to the producer, other characteristics are as follows: d isomer  =  1.4%; relative viscosity  =  3.94; and residual monomer  =  0.14%. ZnO nanofiller was supplied by Umicore Zinc Chemicals (Umicore Zinc Chemicals, Brussels, Belgium) as Zano 20. According to the supplier, these nanoparticles are characterized by a spherical morphology, having diameters of 20–60 nm. Joncryl^®^ 4370 was supplied by BASF (Ludwigshafen am Rhein, Germany) and used here as a PLA chain extender with high-epoxy functionality. According to its technical sheet, this product has a *M*_w_ = 6500–7000, epoxy-equivalent by weight 270–300 g/mol and a glass transition temperature of about 61 °C. It will be known as “Joncryl” hereafter, or it will be abbreviated as “CE”. Ultranox 626A (Bis (2,4-di-*t*-butylphenyl) pentraerythritol diphosphite) supplied by Brenntag NV (described below as U626) was selected as a thermal stabilizer and used at a preferred percentage of 0.25 wt % in all PLA compositions. Throughout this contribution, all percentages are given as weight percentage (wt %).

### 2.2. PLA Films Preparation and Antimicrobial Properties Analysis

Before processing, PLA was dried at 65 °C for 48 h using a drying oven with recirculating hot air. To minimize the water content for melt-blending with PLA, the ZnO nanofiller and Joncyl CE were dried beforehand using similar conditions. PLA granules were dry-mixed with the nanofiller and additives (CE and U626) using a Zeppelin Henschel intensive mixer. Then, PLA masterbatches containing 20% of ZnO, 1% of Joncryl, and 25% of U626 were prepared by melt-compounding using a Leistritz ZSE 18 HP-40D twin-screw extruder screw diameter (D) = 18 mm, L (length/D ratio = 40) and the following processing conditions with a throughput of 2 kg/h, screw speed = 200 rpm, the temperatures of extrusion on the heating zones adapted to the rheological characteristics of PLA blends, e.g., extrusion temperatures on heating zones: Z1 = 130 °C; Z2 = 140 °C; Z3 = 140 °C; Z4 = 150 °C; Z5 = 160 °C; Z6 = 170 °C; Z7 = 160 °C, while the temperature of the molten polymer before the extrusion of strands at the die was kept around 150 °C.

Masterbatch granulates were used for the preparation of tri-layered films. Tri-layered thin films were processed on semi-pilot extrusion line consisting of three single-screw extruders connected to a co-extrusion tri-layered film die (length 300 mm, thickness 1 mm). The core film of 50 µm thickness was carried out by the principal single-screw extruder (Collin, screw diameter 30 mm, L/D ratio 30) operating at 150 rpm with the following temperature profile (150, 160, 170, 180, 190, 190, 170, 170 °C from the hopper to the extruder exit) using pure PLA 4032D. Thin shells were carried out by two other peripheric single-screw extruders (Collin, screw diameter 25 mm, L/D ratio 25) operating under the same conditions as the main extruder. The previously synthesized masterbatch containing 20% of ZnO was diluted with pure, dried PLA reaching surrounding layers of 50 µm thickness each and containing 1% of ZnO. Tri-layered PLAZnO1%/PLA/PLAZnO1% extruded films of 150 µm thickness and were continuously cooled to 50 °C with a calendaring unit.

The PLA film samples devoid of nano-ZnO (control samples) and PLA film samples containing ZnO nanoparticles have been cut into square shapes (3 cm × 3 cm). The activity of PLA films against microorganisms was carried out according to ASTM E 2180-01 standard before and after Q-SUN and UV-A irradiation [32].

### 2.3. Accelerated UV-A and Q-SUN Irradiation

The PLA film samples devoid of nano-ZnO (control samples) and PLA film samples containing ZnO nanoparticles were cut into rectangle shapes (23.5 cm × 7.0 cm and 26.0 cm × 2.5 cm) respectively. The films were placed directly into a UV-A accelerated weathering tester with 1.55 W/m^2^ (QUV/spray, Q-LAB) and into Q-SUN accelerated xenon test chamber with 1.5 W/m^2^ (Model Xe-2, Q-LAB) and irradiated 24 h [33].

### 2.4. Fourier Transform Infrared Analysis 

Fourier transform infrared (FT-IR) spectrum of the PLA film samples devoid of nano-ZnO (control samples) and PLA film samples containing ZnO nanoparticles (before and after UV-A and Q-SUN irradiation) was measured using FT-IR spectroscopy (Perkin Elmer Spectrophotometer, Spectrum 100, Waltham, MA, USA), operated at a resolution of 4 cm^−1^, over four scans. PLA films have been cut into square shapes (2 cm × 2 cm) and introduced into the ray-exposing stage. The spectrum was recorded at a wavelength of 650–4000 cm^−1^.

### 2.5. Mechanical Analysis

A tensile test of PLA film samples containing ZnO nanoparticles was then carried out in order to examine their mechanical properties before and after Q-SUN and UV-A irradiation. A tensile test was carried out according to the ASTM (PN-EN ISO 527-3:1998) [34] standard by using a Zwick/Roell Z 2.5 (Wrocław, Poland).

### 2.6. Statistical Analysis

For mechanical parameter evaluation, statistical significance was determined using an analysis of variance (ANOVA) followed by a Duncan’s Multiple Ranges test. Non-parametric Kruskal-Wallis ANOVA and median tests, followed by multiple comparisons of mean ranks for all groups, was used to determine significant differences between numbers of bacterial cells. The values were considered as significantly different when *p* < 0.05. All analyses were performed with Statistica version 13.1 (StatSoft Polska, Kraków, Poland).

## 3. Results

### 3.1. The Activity of PLA Films against Chosen Microorganisms

**Figure 1:**
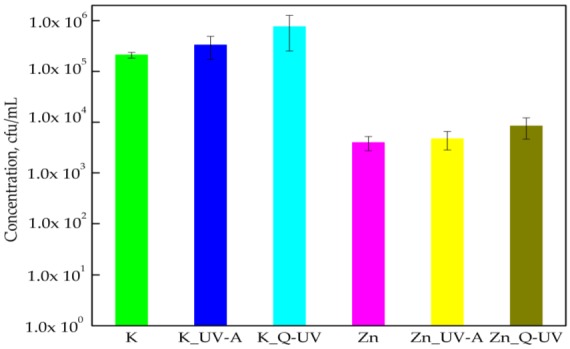
The influence of PLA films containing ZnO nanoparticles on *S*. *aureus* growth. K—PLA film; K_UV-A irradiated PLA film; K_Q-UV irradiated PLA film; Zn—PLA film containing incorporated ZnO nanoparticles; Zn_UV-A irradiated film, containing incorporated ZnO nanoparticles; and Zn_Q-UV irradiated film, containing incorporated ZnO nanoparticles.

This research paper demonstrated that PLA films did not inhibit the growth of *S*. *aureus*, which was confirming a previous article [7]. The PLA films with incorporated ZnO nanoparticles reduced the number of viable *S*. *aureus* cells, but only to a very small degree (Figure 1). The number of the bacteria decreased from 2.10 × 10^5^ cfu/mL to 3.96 × 10^3^ cfu/mL. The differences between the numbers of viable bacterial cells were found to be statistically significant (*p* < 0.05). ZnO nanoparticles incorporated into the PLA matrix were considered bacteriostatic due to their ability to reduce the growth of *S. aureus* cells. The previous study conducted by the authors confirmed that in contrast to films containing incorporated nano-ZnO, films covered with methyl-hydroxy-propyl-cellulose coatings with ZnO nanoparticles destroyed these microorganisms completely and could be considered bactericidal [6]. Microorganisms, such as bacterial strains, are generally characterized by their cell membrane, cell wall, and cytoplasm. The cell wall is composed mostly of a peptidoglycan layer, which maintains the osmotic pressure of the cytoplasm. Gram-positive bacteria have one cytoplasmic membrane with a multilayer of peptidoglycan polymer. ZnO nanoparticles’ size within such ranges could pass through the peptidoglycan and, hence, are highly susceptible to damage. The differences between the activities of the films with ZnO nanoparticles that were introduced into the polymer matrix and active coatings with nano-ZnO may be caused by the fact that a migration of ZnO from coatings was simpler than from a polymer film [3].

Accelerated UV-A irradiation did not influence the activity of PLA films with nano-ZnO against *S. aureus*, and this paper confirms previous findings [6]. It was noted that the number of bacterial cells increased in films that were irradiated with Q-SUN (Zn_Q-UV) radiation (Figure 1). An increase in the number of the *S*. *aureus* was observed from 2.10 × 10^5^ to 7.62 × 10^5^ cfu/mL. The differences between the numbers of bacteria were found to be significant, which was confirmed by a Kruskal-Wallis test. The ability of nano-ZnO to block UV-irradiation caused that the bacteriostatic effect of PLA films with incorporated ZnO nanoparticles after accelerated UV aging was observed in both cases.

**Figure 2:**
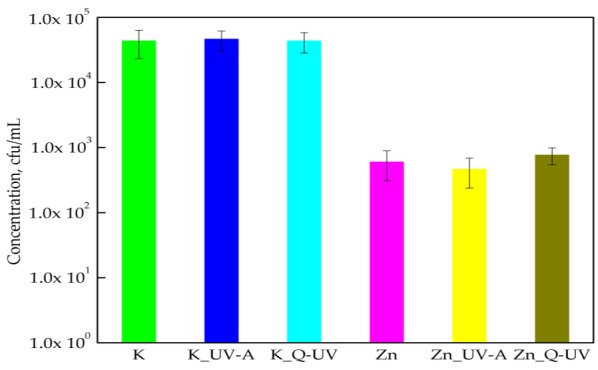
The influence of PLA films containing ZnO nanoparticles on *B*. *atrophaeus* growth. K—PLA film; K_UV-A irradiated PLA film; K_Q-UV irradiated PLA film; Zn—PLA film containing incorporated ZnO nanoparticles; Zn_UV-A irradiated film, containing incorporated ZnO nanoparticles; and Zn_Q-UV irradiated film, containing incorporated ZnO nanoparticles.

Figure 2 shows the susceptibility assay of *B*. *atrophaeus* isolated with respect to active PLA films with nano-ZnO in their matrix. PLA films devoid of nanoparticles were not active against *B*. *atrophaeus* strain. Completely opposite results were found for films containing ZnO nanoparticles incorporated into the polymer matrix. Bacteria numbers decreased from 4.34 × 10^4^ cfu/mL to 6.00 × 10^2^ cfu/mL. It was demonstrated that *B*. *atrophaeus* cells were significantly sensitive towards the active films (*p* < 0.05). The effect of the Q-SUN and UV-A irradiation on the antibacterial properties of films with nano-ZnO was not observed. The amount of viable bacterial cells only slightly increased (below 1 log) for films with nano-ZnO irradiated with Q-SUN (from 6.00 × 10^2^ cfu/mL to 7.66 × 10^2^ cfu/mL). Statistical analysis showed that the differences between the numbers of *B*. *atrophaeus* cells were not significant (*p* > 0.05). A comparison of sample activity should be mentioned here, as the bacteriostatic effect of the films before and after accelerated UV-aging was noted at both points.

**Figure 3:**
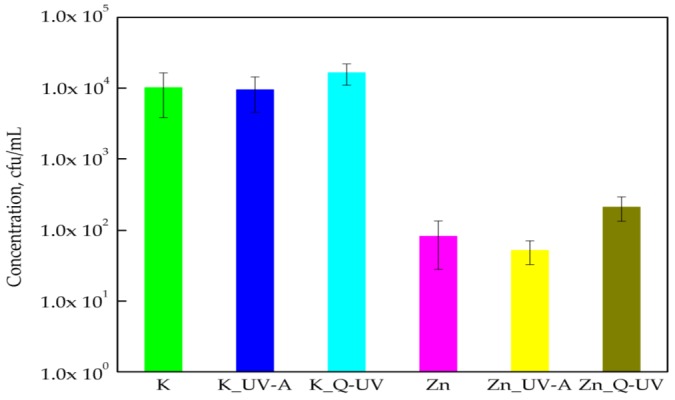
The influence of PLA films containing ZnO nanoparticles on *B*. *cereus* growth. K—PLA film; K_UV-A irradiated PLA film; K_Q-UV irradiated PLA film; Zn—PLA film containing incorporated ZnO nanoparticles; Zn_UV-A irradiated film, containing incorporated ZnO nanoparticles; and Zn_Q-UV irradiated film, containing incorporated ZnO nanoparticles.

The results of this research determined that PLA films were not active against *B*. *cereus*, with the bacterial cells exhibiting sensitivity towards PLA films containing ZnO nanoparticles. A reduction in the amount of viable *B*. *cereus* was noted from 1.01 × 10^4^ to 8.20 × 10^1^ cfu/mL (Figure 3). Statistical analysis confirmed that differences between the numbers of Gram-positive bacilli were significant (*p* < 0.05). UV-A irradiation did not have a significant effect on the antibacterial activity of films containing ZnO nanoparticles (*p* > 0.05). In contrast to UV-A irradiation, accelerated Q-SUN irradiation decreased the antibacterial activity of PLA films containing nanoparticles. A 1 log increase in the amount of *B*. *cereus* for active film samples after Q-SUN accelerated aging was noted. The differences between the numbers of bacteria were not significant, which was confirmed by a Kruskal-Wallis test (*p* > 0.05). To summarize, results obtained for *B. cereus* were similar to those obtained for *B. atrophaeus*. The active films containing ZnO nanoparticles did not inhibit the growth of the Gram-positive bacilli, but they did reduce bacteria numbers. Marra et al. [5] mentioned that ZnO particles exhibited antimicrobial activity against bacteria that were resistant to high temperatures and pressure. It is tempting to suggest that nanoparticles are not active against endospores that are resistant to high temperature and chemicals.

The results of this research demonstrated that PLA films had no influence on the decrease of the number of *E. coli* viable cells, which was emphasized in a previous study [7]. A 1 log decrease in the number of Gram-negative bacteria after a 24 h incubation with PLA films together with an active agent was noted. A Kruskal-Wallis test confirmed that the reduction was significant. Superior results were obtained for *S. aureus*, *B. cereus*, and *B. atrophaeus*, than for *E. coli*. The reason for the higher reduction of Gram-positive bacteria may be that the bacteria wall is composed of two cell membranes, an outer membrane and a plasma membrane with a thin 7–8 nm layer of peptidoglycan [5]. ZnO nanoparticles’ size within such ranges can readily pass through the peptidoglycan. The thinner layer of peptidoglycan makes Gram-negative cells more resistant to nanoparticles than Gram-positive bacteria, though contradictory results were obtained by Arakha et al. [18]. The authors analyzed the antimicrobial properties of the positively-charged ZnO nanoparticles against *E. coli* and *B.* *subtilis* isolates. Gram-negative bacteria were found to be more sensitive towards ZnO nanoparticles than Gram-positive bacteria. The hypothesis of the authors is that since *E. coli* is a Gram-negative microorganism, it possesses more negative surface potential than *B. subtilis*, which is a Gram-positive bacteria. The positively-charged ZnO nanoparticles (p-nanoZnO) take the interfacial potential at the p-nanoZnO/bacteria interface to neutral, suggesting surface neutralization by the respective surface functional groups present on the interacting partner. It was noted that as a result of the neutralization, any energy released was possibly either utilized in the production of ROS or membrane surface tension, or both. The authors suggest that the generation of ROS on the surface of ZnO nanoparticles plays an important role in antimicrobial activity. It was observed that the production of more ROS leading to more cell death was higher for *E. coli* than *B. subtilis*. Together, the bio-nano interfacial potential resulted in a surface tension generating high lateral stress in the membrane leading to irreversible membrane damage via membrane blebbings or rupture. Taken altogether, both biophysical and antimicrobial data obtained from the study has led the authors to hypothesize that the interfacial potential at the p-nanoZnO/bacteria interface was largely responsible for the antimicrobial propensity of ZnO nanoparticles.

**Figure 4:**
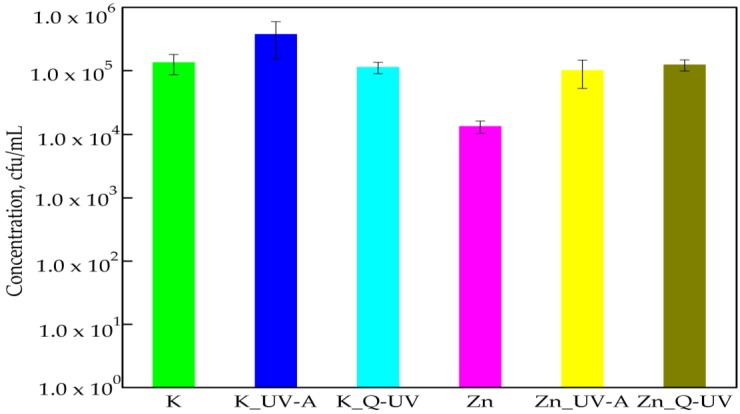
The influence of PLA films containing ZnO nanoparticles on *E*. *coli* growth. K—PLA film; K_UV-A irradiated PLA film; K_Q-UV irradiated PLA film; Zn—PLA film containing incorporated ZnO nanoparticles; Zn_UV-A irradiated film, containing incorporated ZnO nanoparticles; and Zn_Q-UV irradiated film, containing incorporated ZnO nanoparticles.

Figure 4 shown the effect of Q-SUN and UV-A aging on the antibacterial activity of films with ZnO nanoparticles. The number of bacterial cells increased from 1.34 × 10^4^ cfu/mL to 9.98 × 10^4^ cfu/mL for UV-A irradiation and to 1.23 × 10^5^ cfu/mL for Q-SUN irradiation. The differences between the numbers of *E. coli* cells were found to be significant (*p* < 0.05). Silvestre et al. [16] did not confirm the results of this work. The authors observed that the addition of ZnO nanoparticles into the polymer matrix imparted improvements on the UV resistance of polymer film to ultraviolet irradiation. They determined that the packaging material exhibited significant antibacterial activity against *E. coli*, which depended on exposure. 

Contrary to results obtained in [6], a previous study demonstrated that the growth of Gram-negative microorganisms after 24 h incubation with a film covered with the coatings containing ZnO nanoparticles was not noted. Additionally, the effect of accelerated UV aging on the antibacterial activity of coatings containing nanoparticles was also not observed.

**Figure 5:**
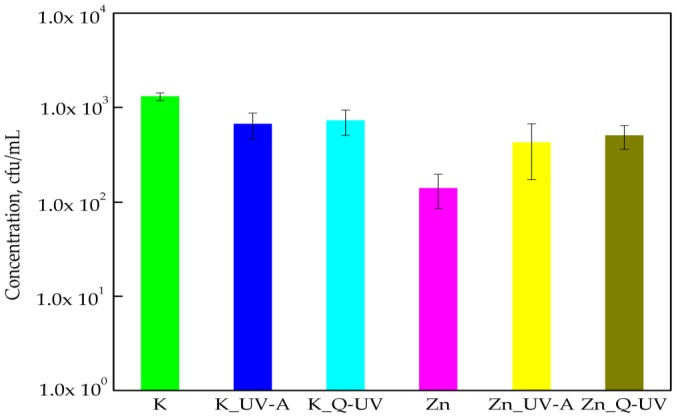
The influence of PLA films containing ZnO nanoparticles on *C*. *albicans* growth. K—PLA film; K_UV-A irradiated PLA film; K_Q-UV irradiated PLA film; Zn—PLA film containing incorporated ZnO nanoparticles; Zn_UV-A irradiated film, containing incorporated ZnO nanoparticles; and Zn_Q-UV irradiated film, containing incorporated ZnO nanoparticles.

The results of this research paper determined that PLA films did not influence the growth of *C*. *albicans* cells compared to PLA films containing ZnO nanoparticles. Nano-ZnO incorporated in a PLA matrix decreased the amount of *C*. *albicans*. A 1 log reduction in the amount of the viable cells was noted. Statistical analysis showed nano-ZnO had a significant effect on the activity of (K) PLA films (*p* < 0.005). This was also noted in a previous study [6] and showed that *C*. *albicans* were sensitive towards ZnO nanoparticles. It was interesting [35,36] that the size of the cellular membrane pores (with diameters measured in nanometers) were small enough to allow the nanoparticles to cross the cell membranes [6,30,35]. Accelerated UV-A irradiation did not deactivate the activity against microorganisms of coatings containing ZnO [6], which was noted in a previous study. In contrast to this earlier study, the results of these experiments demonstrated that UV-A the irradiation of films containing nano-ZnO caused a 1 log increase in the number of *C*. *albicans* cells (Figure 5), though this was not significant (*p* > 0.05), and this was as confirmed by a Kruskal-Wallis test. A 1 log increase in the amount of *C*. *albicans* cells was observed in films containing incorporated ZnO nanoparticles after aging with Q-UV. This meant that Q-SUN aging decreased the antimicrobial properties of the PLA films. A moderately similar result was also obtained in a previous study [6].

### 3.2. Fourier Transform Infrared Spectroscopy

**Figure 6:**
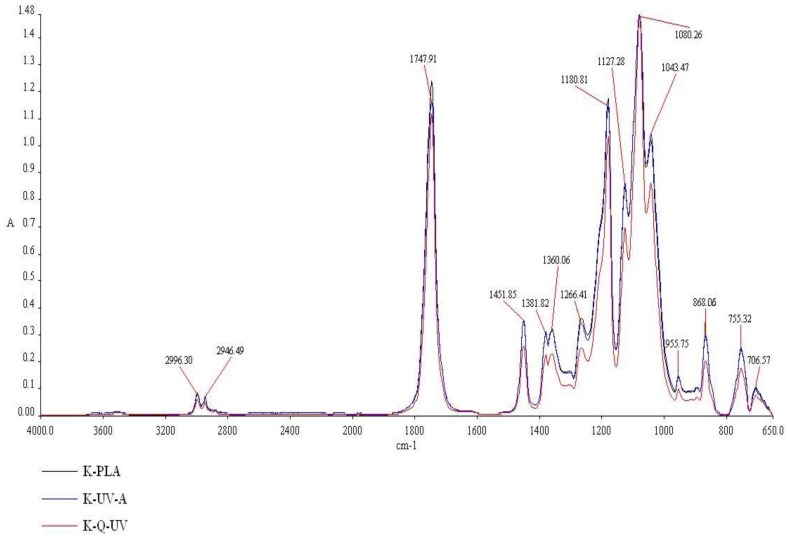
The FT-IR spectra of PLA films. K-PLA—PLA film; K-UV-A—irradiated PLA film; K-Q-UV—irradiated PLA film.

**Figure 7:**
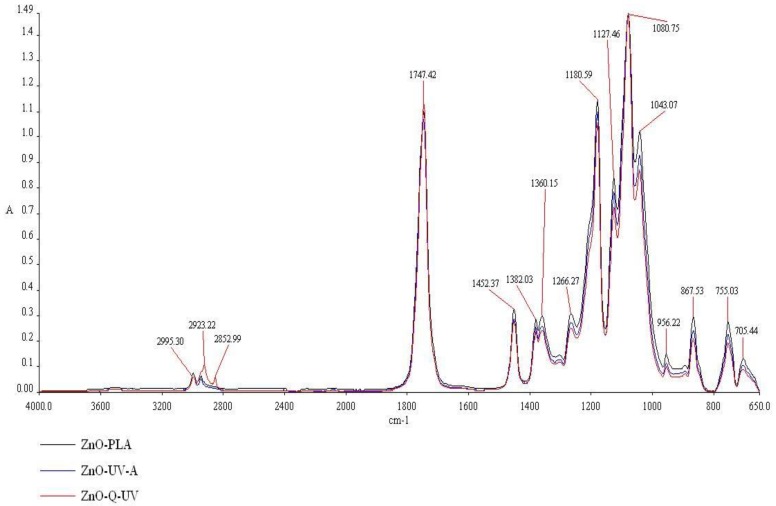
The FT-IR spectra of PLA films containing ZnO nanoparticles. ZnO-PLA—PLA film containing incorporated ZnO nanoparticles; ZnO-UV-A—irradiated film, containing incorporated ZnO nanoparticles; and ZnO-Q-UV—irradiated film, containing incorporated ZnO nanoparticles.

The effect of UV-A and Q-SUN aging on PLA films with ZnO nanoparticles or devoid of nanoparticles may be observed using Fourier transform infrared (FT-IR) spectroscopy. The properties that may influence the absorption peak and band positions are the chemical composition of the structure and the morphology of the films [6,22]. It was determined in this research that there were no significant changes in the chemical composition and morphology of PLA films before (K) and after UV-A (K_UV-A) and Q-SUN aging (K_Q-UV). These results confirmed that accelerated aging did not have an influence on PLA films (Figure 6). It was determined that there were four regions viewed in the FT-IR spectroscopy, extending over (1) ranges from 3600 to 2800 cm^−1^; (2) ranges from 1800 to 1600 cm^−1^; (3) ranges from 955 to 650 cm^−1^; and (4) ranges from 1460 to 1000 cm^−1^. In the case of a 1451.85 cm^−1^ peak, a 1381.82 cm^−1^ peak, a 1360.06 cm^−1^ peak, a 1266.41 cm^−1^ peak, a 955.75 cm^−1^ peak, an 868.06 cm^−1^ peak, and a 755.32 cm^−1^ peak, the intensity of peaks decreased after irradiation. The effect of UV-A and Q-UV irradiation on films containing ZnO nanoparticles was not noted (Figure 7). The previous study [6] demonstrated that accelerated UV-A and Q-SUN irradiation had no influence on the chemical composition of a polymer coating with nano-ZnO. The results of the study have also shown that Q-SUN aging altered the chemical composition of the MHPC coating that covered the polymer film. It was observed [6,30] that ZnO nanoparticles shielded the coating against Q-SUN aging.

### 3.3. Mechanical Analysis

The mechanical analysis of the PLA containing incorporated ZnO nanoparticles showed that an elongation at break (measured in machine direction) decreased after UV-A or Q-UV irradiation, but this was a slight change (Table 1). The differences between elongation at break values were not significant, as confirmed by a Duncan test (*p* > 0.05). The elongation at break values obtained with transverse direction showed that accelerated UV-A and Q-UV irradiation did not have any influence on them, which was confirmed by statistical analysis (*p* < 0.05). F_max_ (maximum tensile strength) values also altered slightly, with the differences between F_max_ before and after irradiation found to not be significant (*p* > 0.05). Similar results were obtained in the case of F at break (Table 1).

## 4. Discussion

ZnO nanoparticles are described as a functional, strategically-promising material with a broad range of applications and advantages, such as low cost and white appearance [7,35]. ZnO nanoparticles have unique antibacterial and antifungal properties and are non-toxic to human cells [35,36,37,38,39]. Many studies have proved that fungal strains, such as *Aspergillus flavus*, *A*. *niger*, and *C. albicans*, are sensitive to ZnO nanoparticles [35,36,37]. The antibacterial activity of the nanoparticles against Gram-positive strains, such as *S*. *aureus*, *B*. *cereus*, *B*. *atrophaeus*, and Gram-negative strains, such as *E*. *coli* and *P. aeruginosa*, was also noted [6,7,35,36,37,38,39,40,41,42]. Esmailzadeh et al. [27] and Gandhi et al. [43] found Gram-positive bacteria to be more sensitive to ZnO nanoparticles than Gram-negative bacteria, and those results were confirmed by this research, though contradictory results were obtained by Sharma et al. [44]. The authors proved that *S*. *aureus* cells were more resistant towards ZnO nanoparticles than *E*. *coli*. Previous studies [5,6] indicated that ZnO nanoparticles can be used as active agents in MHPC carriers to coat packaging materials and that active coatings exhibited antimicrobial activity against *S*. *aureus*, *B. cereus*, *P. aeruginosa*, and *E*. *coli* cells, where all of the bacteria were killed. In contrast to previous results that demonstrated the bactericidal effect of the coatings, the results of this study have shown that ZnO nanoparticles incorporated into a biopolymer matrix displayed bacteriostatic qualities. It has been shown that active films did not kill bacteria, but did reduce the numbers of *S*. *aureus*, *B. cereus*, *B. atrophaeus*, and *E*. *coli* cells. It should be mentioned that better results were obtained for *S. aureus*, *B. cereus*, and *B. atrophaeus* than for *E. coli*. The difference between the coatings containing ZnO nanoparticles [5,6] and the films containing ZnO nanoparticles was that the coatings inhibited the growth of bacteria completely, while the films only reduced the number of viable cells. It is tempting to suggest that a migration of nano-ZnO from coatings would be simpler than from a polymer matrix. The aim of this research was to investigate the effect of accelerated UV-A and Q-SUN irradiation on the antimicrobial activity of PLA films with ZnO nanoparticles. A second aim was to investigate the capability of ZnO nanoparticles to protect their own antimicrobial properties. The results demonstrated that accelerated UV-A and Q-UV aging did not have any significant effect on the activity of PLA films containing ZnO nanoparticles against two Gram-positive strains: *S. aureus* and *B. atrophaeus*. Only Q-UV aging decreased the number of viable *B. cereus* cells, though only in a minor way. It is known [3] that ZnO nanoparticles possess a high optical absorption in the UV-A (315–400 nm) and UV-B (280–315 nm) regions, beneficial in antibacterial response and used as a UV protector. These results were confirmed by previous studies [6] demonstrating that accelerated aging had no effect on the deactivation of the antimicrobial properties of films covered with nano-ZnO active coatings. The results of this research demonstrated that accelerated UV-A and Q-UV irradiation decreased the activity of films with ZnO nanoparticles against *E. coli* and *C. albicans*, though the results shown here cannot be compared with those demonstrated in previous studies [6], which proved that the growth *E. coli* cells was not observed after contact with nano-ZnO irradiated coatings. A reduction in the number of *C. albicans* cells was noted, but only for coatings with nanoparticles irradiated with Q-UV.

The mechanical properties of the polymer film can be improved when ZnO nanoparticles are dispersed in the matrix. An improvement in mechanical behavior is expected, which could be due to the correct dispersion of ZnO nanoparticles in the polymer matrix. Venkatesan et al. [42] demonstrated that the tensile strength and elongation of different types of polymer films increased significantly by incorporating ZnO nanoparticles into the polymer matrix. Similar results were obtained by Chu et al. [45]. The authors ascertained that the mechanical properties of PLA were enhanced on the incorporation of nano-ZnO into a PLA matrix. This positive change may have occurred due to the addition of nanoparticles to the polymer matrix resulting in an increase in ductile properties, which would also result in changes in the crystallinity of the polymer [44,45]. The results of this study indicate that accelerated UV-A and Q-UV irradiation did not influence the mechanical properties of PLA films containing incorporated ZnO nanoparticles. The PLA films were found to be resistant to UV aging and it is tempting to suggest that the UV shielding ability of the ZnO nanoparticles incorporated in PLA films protected the PLA matrix during accelerated irradiation.

## 5. Conclusions

The PLA films containing incorporated ZnO nanoparticles were active against *S. aureus*, *B. atrophaeus*, *B. cereus*, *E. coli*, and *C. albicans* strains.

Accelerated UV aging had no negative effect on the activity of the film containing nano-ZnO against Gram-positive bacteria, but it had an influence on the activity of Gram-negative cells and *C. albicans*. Q-SUN irradiation decreased the antimicrobial effect of films with incorporated nanoparticles against *B. cereus*.

Accelerated UV-A and Q-UV irradiation had no influence the mechanical properties of PLA films containing incorporated ZnO nanoparticles.

## Figures and Tables

**Table 1:** The mechanical properties of PLA films containing ZnO nanoparticles before and after irradiation.

Sample		F_max_	F at Break	Elongation at Break
		N	N	%
Not irradiated	Machine direction	143.13 ± 15.23	26.80 ± 4.50	109.26 ± 3.87
	Transverse direction	97.93 ± 14.91	22.60 ± 3.66	102.36 ± 0.67
Irradiated with UV-A	Machine direction	140.13 ± 22.59	24.68 ± 5.92	106.12 ± 1.20
	Transverse direction	105.78 ± 7.64	18.78 ± 2.63	103.05 ± 0.44
Irradiated with Q-UV	Machine direction	141.50 ± 18.07	27.37 ± 2.91	105.69 ± 2.04
	Transverse direction	93.43 ± 10.13	21.40 ± 1.46	102.36 ± 0.05
